# Phosphate Binding with Sevelamer Preserves Mechanical Competence of Bone Despite Acidosis in Advanced Experimental Renal Insufficiency

**DOI:** 10.1371/journal.pone.0163022

**Published:** 2016-09-22

**Authors:** Jarkko Jokihaara, Ilkka H. Pörsti, Harri Sievänen, Peeter Kööbi, Pekka Kannus, Onni Niemelä, Russell T. Turner, Urszula T. Iwaniec, Teppo L. N. Järvinen

**Affiliations:** 1 Department of Hand and Microsurgery, Tampere University Hospital, Tampere, Finland; 2 Center for Hip Health and Mobility, Department of Orthopaedics, University of British Columbia, Vancouver, BC, Canada; 3 Medical School, University of Tampere, Tampere, Finland; 4 Bone Research Group, UKK-Institute, Tampere, Finland; 5 Department of Laboratory Medicine, Seinäjoki Central Hospital Laboratory, Seinäjoki, Finland; 6 Skeletal Biology Laboratory, School of Biological and Population Health Sciences, Oregon State University, Corvallis, OR, United States of America; 7 Department of Orthopaedics and Traumatology, University of Helsinki, Helsinki, Finland; University of Florida College of Medicine, UNITED STATES

## Abstract

**Introduction:**

Phosphate binding with sevelamer can ameliorate detrimental histomorphometric changes of bone in chronic renal insufficiency (CRI). Here we explored the effects of sevelamer-HCl treatment on bone strength and structure in experimental CRI.

**Methods:**

Forty-eight 8-week-old rats were assigned to surgical 5/6 nephrectomy (CRI) or renal decapsulation (Sham). After 14 weeks of disease progression, the rats were allocated to untreated and sevelamer-treated (3% in chow) groups for 9 weeks. Then the animals were sacrificed, plasma samples collected, and femora excised for structural analysis (biomechanical testing, quantitative computed tomography).

**Results:**

Sevelamer-HCl significantly reduced blood pH, and final creatinine clearance in the CRI groups ranged 30%-50% of that in the Sham group. Final plasma phosphate increased 2.4- to 2.9-fold, and parathyroid hormone 13- to 21-fold in CRI rats, with no difference between sevelamer-treated and untreated animals. In the femoral midshaft, CRI reduced cortical bone mineral density (-3%) and breaking load (-15%) (p<0.05 for all versus Sham), while sevelamer increased bone mineral density (+2%) and prevented the deleterious changes in bone. In the femoral neck, CRI reduced bone mineral density (-11%) and breaking load (-10%), while sevelamer prevented the decrease in bone mineral density (+6%) so that breaking load did not differ from controls.

**Conclusions:**

In this model of stage 3–4 CRI, sevelamer-HCl treatment ameliorated the decreases in femoral midshaft and neck mineral density, and restored bone strength despite prevailing acidosis. Therefore, treatment with sevelamer can efficiently preserve mechanical competence of bone in CRI.

## Introduction

Efficient control of hyperphosphatemia is a cornerstone in the treatment of chronic kidney disease-mineral bone disorder (CKD-MBD) [1–5]. If dietary phosphate restriction is not sufficient, the control of hyperphosphatemia in CKD patients is often accomplished by the use of oral calcium salts as phosphate binders [6]. However, excess calcium intake may predispose to hypercalcemia, soft-tissue calcification, and increase the risk of adynamic bone disease and bone fragility [2–4, 7]. As an alternative approach, the non-calcium containing polymer sevelamer is an effective phosphate-binder [8–13].

In a 2-year study with haemodialysis patients, sevelamer treatment prevented the decrease in trabecular bone density in thoracic vertebrae [8], while in peritoneal dialysis patients, 8-month sevelamer treatment improved skeletal changes of secondary hyperparathyroidism [9]. In experimental chronic renal insufficiency (CRI), sevelamer treatment ameliorated the histomorphometric changes of femoral bones in rats subjected to adenine diet-induced renal damage [12], while in a murine model of metabolic syndrome and CRI with low bone turnover, sevelamer treatment reversed the adynamic bone disorder in these animals [13]. However, sevelamer hydrochloride (HCl) increases dietary acid load and this may predispose to acidosis, with the potential to exacerbate secondary hyperparathyroidism and renal bone disease [14, 15]. For these reasons, sevelamer-HCl has been replaced by sevelamer carbonate [13]. However, at the time when our study was conducted the manufacturer could not provide sevelamer carbonate for experimental purposes.

While sevelamer treatment has provided benefits to bone density and histology in CRI, information about the influence of sevelamer on the mechanical competence of bone is lacking. Since the principal task of bones is to bear skeletal loads without breaking [16, 17], we chose an organ-level approach to explore the influences of sevelamer. Due to the divergent effects of CRI on cortical and trabecular bone compartments [18, 19], we examined changes in three structurally distinct femoral regions: diaphysis (essentially cortical bone), distal metaphysis (substantial trabecular compartment), and neck (both cortical and trabecular structures) [16, 17, 19–21]. Rats were subjected to 5/6 nephrectomy, and after 14 weeks of disease progression, treated with 3.0% sevelamer-HCl for 9 weeks, to test the hypothesis whether this phosphate binder can beneficially influence the mechanical competence of bone.

## Methods

### Ethics Statement

The study design was approved by Tampere University Animal Experimentation Committee, and Provincial Government of Western Finland, Department of Social Affairs and Health, Finland. The investigation conforms to the Guide for the Care and Use of Laboratory Animals published by the US National Institutes of Health. As high mortality rates (>80%) had been previously observed in experiments where 5/6 nephrectomized rats were treated with sevelamer-HCl for 6 months [22], the Animal Experimentation Committee advised us to apply a protocol with premature termination of the study in individual rats when necessary (see criteria below).

### Experimental Design

**Fig 1:**
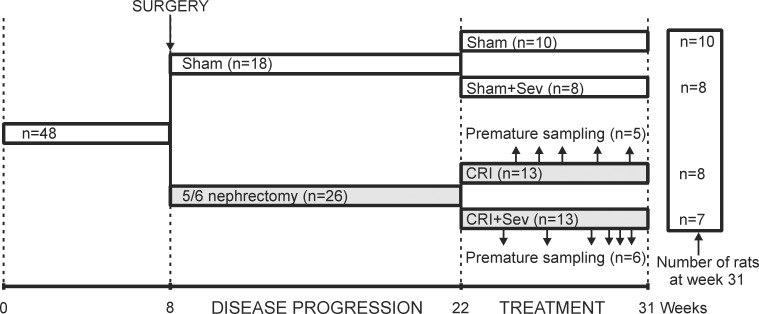
The flowchart of the study. Sham, group subjected to renal decapsulation; CRI, chronic renal insufficiency group subjected to 5/6 nephrectomy; Sev, 3.0% sevelamer-HCl in chow. Four rats were euthanized because of deteriorated general condition during the immediate postoperative period. Small arrows during the treatment period denote premature sampling of individual rats due to deteriorated physical state (see Methods).

Forty-eight 8-week old male Sprague-Dawley rats were subjected to surgical 5/6 nephrectomy (n = 30) or sham-operation (renal decapsulation, n = 18) [23–26]. Postoperative pain was relieved with 0.2 mg/kg buprenorphine (subcutaneously; Reckitt & Colman, Hull, UK) 3 times a day during the first 3 postoperative days. The chow contained 0.9% calcium and 0.8% phosphate (Lactamin R34, AnalyCen, Sweden) during 14 weeks of disease progression. Then urine was collected in metabolic cages, and plasma samples were drawn from the tail vein. The 5/6 nephrectomized rats (n = 26) were randomized to two groups with equal body weights, 24-hour urine outputs, plasma creatinines, and amounts of kidney tissue removed (Table 1, Fig 1): untreated (CRI, n = 13) and sevelamer-treated rats (CRI+Sev, n = 13). The Sham rats (n = 18) were randomized to untreated (Sham) and sevelamer-treated groups (Sham+Sev). Before the treatment, plasma phosphate concentrations in the sham-operated vs. 5/6 nephrectomized rats were 1.32±0.07 vs. 2.02±0.17 mmol/l, and plasma PTH 56±12 vs. 435±49 pg/ml, respectively (p<0.05 for both). During 9 weeks of treatment, all groups continued on 0.3% calcium and 0.8% phosphate chow (AnalyCen), and 3% sevelamer-HCl (RenaGel, Genzyme, MA, USA) was added to the chow of CRI+Sev and Sham+Sev rats (Fig 1) [12, 27]. All rats were housed 3–4 per cage in an animal laboratory (illuminated 06:00-18:00 h, temperature + 22°C) with free access to water and food [26].

**Table 1:** Gross clinical characteristics and laboratory findings in the study groups.

	Sham n = 10	Sham+Sev n = 8	CRI n = 11–13	CRI+Sev n = 11–13
Animal weight (g)				
Before treatment^1^	447±13	453±7	447±9	440±12
Final^2^	484±14	508±20	455±91^§^	442±69^§^
Femur length (mm)	40.7±1.0	40.8±0.6	40.8±1.0	40.4±1.1
Kidney removed^3^ (g/kg)	-	-	6.37±0.12	6.31±0.13
Creatinine (μmol/l)				
Before treatment^1^	43±5	44±4	85±6^§§§^	89±6^§§§^
Final^2^	50±3	51±4	139±44^§§^	264±77^§§^
Creatinine clearance (ml/min)^4^	2.0±0.2	2.1±0.2	1.1±0.1^§§§^	0.6±0.2^§§§^
Urea (mmol/l)	6.8±0.3	7.2±0.4	26.9±8.4^§§§^	45.8±11.4^§§§^
Phosphate (mmol/l)				
Crude values	1.28±0.07	1.46±0.05	3.03±0.79^§^	3.67±1.07^§^
Creatinine-adjusted values	2.50±0.29	2.67±0.33	3.02±0.26	1.91±0.28^†^
Calcium (mmol/l)	2.34±0.02	2.34±0.03	2.28±0.06	2.40±0.04
PTH (pg/ml)^5^	54±18	106±35	1173±351^§§§^	732±314^§§§^
25OH-D_3_ (nmol/l)	57.2±3.7	45.4±4.4^‡‡^	41.9±3.7^§§§^	28.0±2.3^§§§^^‡‡^
1,25-(OH)_2_D_3_ (pmol/l)	422±17	151±9^‡‡^	85±19^§§§^	41±16^§§§^^‡‡^
FGF-23 (pg/ml)^5^	746±22	696±34	7377±3102^§§§^	11446±8734^§§§^
Blood pH	7.38±0.05	7.23±0.07^‡^	7.25±0.06	7.12±0.04^‡^
Hemoglobin (g/l)	176±3	181±2	156±5^§§§^	144±9^§§§^

Results are mean±SE.

^1^Week 22

^2^week 31

^3^tissue removed in 5/6 nephrectomy

^4^at week 31 CRI rat number was 8 and CRI+Sev rat number was 9 during 24-hour urine collection

^5^statistics from log-transformed values.

PTH, parathyroid hormone; 25OH-D_3_, calcidiol; 1,25-(OH)_2_D_3_, calcitriol; FGF-23, fibroblast growth factor-23.

^§^P<0.05

^§§^P<0.01

^§§§^P<0.001 CRI main effect

^‡^P<0.05

^‡‡^P<0.01 sevelamer main effect

^†^P<0.05 CRI+sevelamer interaction.

### Data Collection and Samples

During the last study week 24-hour urine was collected. At close, the rats were anaesthetized by intraperitoneal urethane (1.3 g/kg), carotid artery was cannulated, and blood samples were drawn [23–26]. Both femora were excised, cleaned and stored at -20°C in sealed freezer bags [23, 24], using a procedure that preserves their mechanical properties [28, 29]. Blood samples could not be obtained from 2 rats in the CRI and 2 rats in the CRI+Sev group.

#### Premature sampling

The general condition (habitual movement, breathing, signs of discomfort and pain) of the study animals was assessed at least 3 times daily. Premature sampling was applied in 5 CRI and 6 CRI+Sev rats (Fig 1) using the following predefined endpoints: 1) fall of body weight below -2.5 standard deviations of the average weight of all CRI rats (CRI, n = 3; CRI+Sev, n = 3), 2) loss of body weight > 100 g/week (CRI, n = 1; CRI+Sev, n = 2), and 3) deteriorated physical state (swelling, CRI, n = 1; shortness of breath, CRI+Sev, n = 1). Importantly, average treatment lengths were similar in the CRI and CRI+Sev groups: 50.0±3.2 and 50.6±2.5 days, respectively. Renal insufficiency was more advanced in the prematurely sampled rats versus those gone through the whole treatment period (S1 Table).

#### Plasma and urine chemistry

Creatinine, urea, phosphate and calcium concentrations were measured using standard clinical chemical methods (Cobas Integra 800 Analyzer, Roche Diagnostics, Basel, Switzerland). Hemoglobin was measured photometrically (Technicon H*2, Technicon Instruments Corporation, Tarrytown, NY, USA), plasma pH using an ion selective electrode (634 pH Analyzer, Ciba Corning Diagnostics, Sudbury, UK), rat intact PTH levels using immunoradiometric assay (Immutopics Inc. San Clemente, CA, USA), and 25OH-D_2_ and 1,25(OH)_2_D_3_ using radioimmunological assays (IDS Inc., Arizona, USA). Plasma fibroblast growth factor-23 (FGF-23) was determined using ELISA (Kinos Inc., Tokyo, Japan) [26, 27, 30].

### Bone Analyses

For measurements, the femora were thawed at room temperature and kept wrapped in saline-soaked gauzes.

#### Peripheral quantitative computed tomography (pQCT)

Femoral cross-sections were analyzed using pQCT (Stratec XCT Research M, software version 5.40B, Stratec Medizintechnik GmbH, Birkenfeld, Germany). Both femora were scanned and the average value was used.

#### Diaphysis

**Fig 2:**
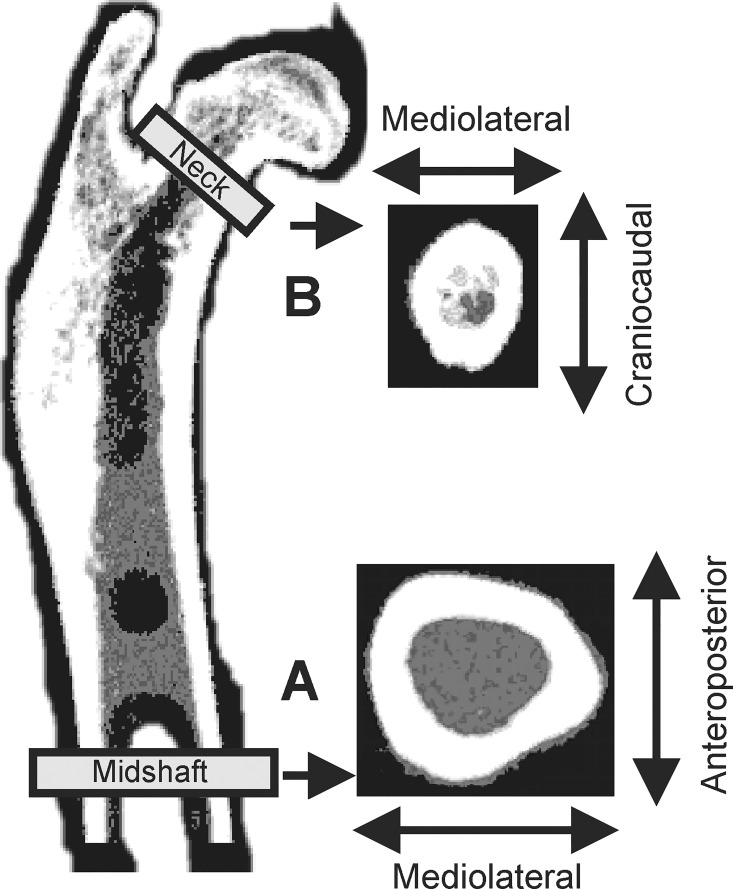
The regions of interest in the femoral bone. (A) midshaft, analyses in mediolateral and anteroposterior directions; (B) neck, analyses in mediolateral and craniocaudal directions.

The femora were scanned at 50% of the femur length (voxel size 0.07 x 0.07 x 0.5 mm^3^, scan speed 3.0 mm/sec). The cortical bone mineral density (cBMD), total bone mineral content (BMC), total cross-sectional area (tCSA), cortical cross-sectional area (cCSA), cortical bone thickness were recorded (Fig 2). The mean square coefficients of variation (CV_rms_) are 0.6% for cBMD, 0.9% for tCSA, and 1.5% for cCSA [31].

#### Femur neck

The femoral neck was scanned twice, repositioning the sample between measurements. The average of tCSA, BMC, bone outside diameters, and total bone mineral apparent density (vBMD; mineral content divided by volume) measurements were used. The CVrms are 2.1% for vBMD and 3.9% for tCSA [31].

### Micro-Computed Tomography (μCT)

The μCT was used for three-dimensional evaluation of trabecular bone architecture at the distal metaphysis from a 1.8 mm section (150 slices) at nominal isotropic voxel size of 12 μm (Scanco μCT 40, Scanco Medical AG) [32]. Volume of interest included only secondary spongiosa. The measurements included: trabecular bone volume fraction (volume of total tissue evaluated occupied by trabecular bone, %), trabecular thickness (μm), trabecular number (1/mm), trabecular separation (μm), connectivity density (number of redundant connections per unit volume, 1/mm^3^; detects defects in trabecular architecture), and structure model index (quantifies the plate versus rod characteristics of trabecular bone; scale 0–3, with 0 representing purely plate-like structures and 3 representing purely rod-like structures) [32].

#### Biomechanical testing

A Lloyd material testing device (LR5K, J.J. Lloyd Instruments, Southampton, UK) was used for the three-point bending of the femoral shafts in anteroposterior (AP) and mediolateral (ML) directions (Fig 2) [33–35]. The right femur was tested in AP and left femur in ML direction. When testing the breaking load (F_max_), the load was applied to the midshaft perpendicularly to the bone axis using a brass crossbar, until failure of the specimen. The CV_rms_ of F_max_ for three-point bending range from 3.8% (ML) to 5.0% (AP) [35].

The proximal part of each specimen was used for femoral neck compression test [33, 34]. Proximal femur was mounted in a fixation device [36], placed under the testing device, and vertical load was applied to the top of the femoral head using a brass crossbar until failure (F_max_). The CV_rms_ of F_max_ for femoral neck compression is 7.6% [34, 35].

#### Statistical analyses

Statistical analyses were performed using one-way and two-way analyses of variance (ANOVA), and the least-significant difference test. If variable distribution was skewed, the Kruskal-Wallis test, supported by Mann-Whitney U-test in the post-hoc analyses, was used, and the p values were corrected with the Bonferroni equation. Spearman correlations were calculated, as appropriate. The results were reported as mean±SEM, and p<0.05 was considered significant.

The stresses in lower extremity bones stem from weight-bearing, bending, and torsional loading produced by muscles [37, 38]. To eliminate the bias arising from comparisons between groups that differ in body weight and size, all data pertaining to bone mechanical competence were normalized by using body weight and femoral length of each rat as covariates in the analyses [31, 39, 40].

## Results

### Systemic Effects of CRI and Sevelamer

At the initiation of therapy, creatinine levels that were measured using an enzymatic method were about two-fold elevated in the CRI versus Sham rats, and further elevation was observed during the treatment period (Table 1). The applied enzymatic method has been shown to fulfil the requirements for plasma creatinine measurements in healthy and diseased rats over a broad concentration range [41]. Experimental CRI resulted in biochemical effects corresponding to stage 3 CKD [2]: creatinine clearance was reduced by ~50%, plasma creatinine was increased 3-fold, and urea level 4-fold (Table 1). The results showed reduced hemoglobin, hyperphosphatemia, increased plasma PTH and FGF-23, and decreased plasma 25OH-D_3_ and 1,25-(OH)_2_D_3_ concentrations. Plasma calcium level was not reduced, which can be attributed to the secondary hyperparathyroidism in CRI. Although CRI resulted in lower final body weight, no effect was observed on longitudinal bone growth, as femoral lengths were similar in all study groups (Table 1).

Although numerically lower indices of renal function were observed in sevelamer-treated versus untreated rats with CRI, final plasma creatinine (*P* = 0.056) and creatinine clearance (*P* = 0.059) were not significantly different between the CRI vs. CRI+Sev groups (Table 1). The correlation (Spearman) between plasma creatinine and phosphate concentrations was strong: 0.93 in all study rats, 0.87 in rats with CRI, 0.81 in the CRI group, and 0.90 in the CRI+Sev group (p<0.001 for all). Sevelamer treatment did not influence plasma calcium, PTH, FGF-23 or blood hemoglobin levels. Unexpectedly, no difference was observed in the *final* plasma phosphate levels between the CRI and CRI+Sev groups. However, given that phosphate metabolism is progressively impaired secondarily to reduced kidney function [4], adjustment of phosphate levels with plasma creatinine uncovered a decrease in plasma phosphate in CRI+Sev versus CRI group (Table 1). Similarly, the creatinine-adjusted PTH-levels in the CRI+Sev versus CRI groups were 242±194 versus 1168±177 pg/ml, respectively (*P* = 0.02). Of note, sevelamer-HCl treatment reduced plasma 25-OH-D_3_ and 1,25-(OH)_2_D_3_ levels and blood pH in both Sham and CRI rats, but was without effect on plasma FGF-23 levels (Table 1).

### CRI, Sevelamer, and the Femoral Diaphysis

#### Bone mineral density and cross-sectional geometry

**Fig 3:**
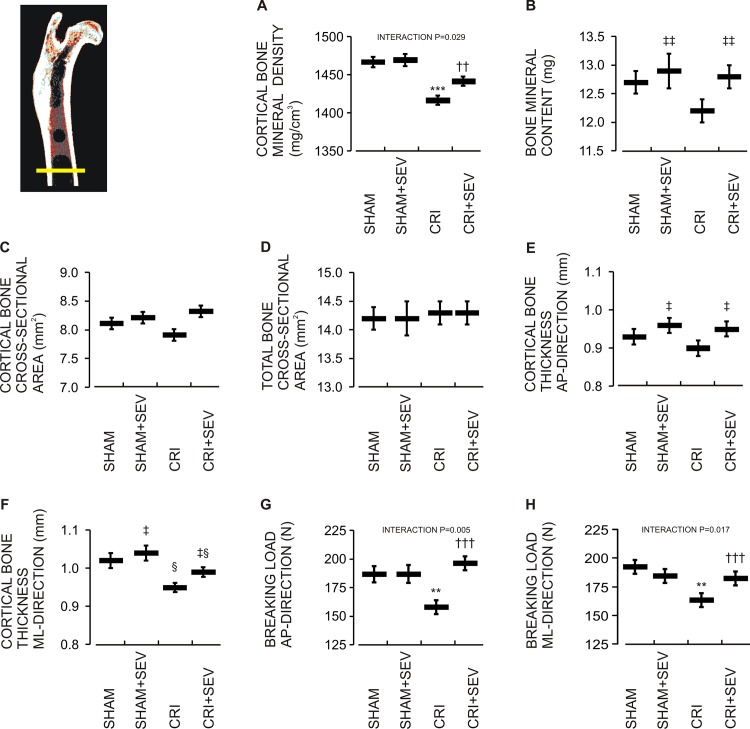
The femoral midshaft. Effect of CRI and sevelamer-HCl treatment on (A) cortical bone mineral density (cBMD), (B) total bone mineral content (BMC), (C) cortical bone cross-sectional area (cCSA), (D) total cross-sectional area (tCSA), (E) cortical thickness in anteroposterior AP direction, cortical thickness in mediolateral direction, (F) breaking load in anteroposterior direction, and (G) breaking load in mediolateral directions (H). Data denotations are mean (line) and SEM (whiskers); ^§^P<0.05 CRI main effect; ^‡^P<0.05, ^‡‡^P<0.01 sevelamer treatment main effect; ***P<0.001 vs. Sham; ^††^P<0.01 vs. CRI.

CRI was associated with decreased cBMD (-3.4%), while sevelamer treatment prevented the decrease in cBMD (+1.8%, P = 0.029 for the interaction, Fig 3A). Neither CRI nor sevelamer treatment influenced cortical bone cross-sectional area (cCSA) or total bone cross-sectional area (tCSA) of the femoral midshaft (Fig 3C and 3D). Bone mineral content (Fig 3B) and midshaft cortical thickness in the AP direction (Fig 3E) were increased by sevelamer when all treated were compared with untreated rats. In the ML direction, CRI was associated with reduced cortical thickness (-6.9%), while this reduction was alleviated by sevelamer (4.2% increase, Fig 3F). Average cortical thickness (AP and ML combined) of the femoral midshaft was not significantly lower in the CRI than the Sham group (-4.6%, p = 0.057), but was 6.0% higher in the CRI+Sev than the CRI group (p<0.01).

#### Fragility

In three-point bending, CRI was associated with clear bone fragility, since midshaft breaking load was decreased in both directions (AP -15.5%, ML -15.1%, Fig 3G and 3H). In rats with CRI, sevelamer prevented the decrease in midshaft breaking load (AP direction +24.1%, ML direction +11.0%, P = 0.005 and P = 0.017 for the interaction, respectively, Fig 3G and 3H).

### CRI, Sevelamer, and the Femoral Neck

**Fig 4:**
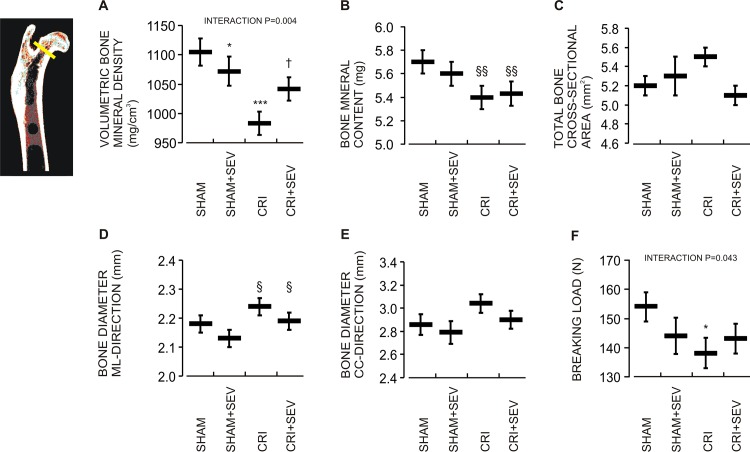
The femoral neck. Effect of CRI and sevelamer-HCl treatment on (A) volumetric bone mineral density (vBMD), (B) total bone mineral content (BMC), (C) total bone cross-sectional area (tCSA), (D) bone diameter in mediolateral (ML) direction, (E) bone diameter in craniocaudal (CC) direction, and (F) breaking load. Data denotations are mean (line) and SEM (whiskers); ^§^P<0.05, ^§§^p<0.01 CRI main effect; *P<0.05, ***P<0.001 vs. Sham; ^†^P<0.05 vs. CRI.

The changes observed in the femoral neck were in good agreement with those observed in the diaphysis. CRI was associated with decreased vBMD (-11.0%), BMC (-5.1%), increased diameter in the ML direction (+3.0%), and decreased breaking load (-10.4%), while no significant changes in the craniocaudal diameter (+6.2%, p = 0.11) and tCSA (+5.7%, p = 0.077) were observed (Fig 4A–4F). In rats with CRI, sevelamer treatment increased vBMD (+6.0%, P = 0.004 for the interaction). The BMC (-4.8%, p = 0.075) and breaking load (-7.1%, p = 0.14) in the CRI+Sev group did not significantly differ from those in the Sham group.

### CRI, Sevelamer, and the Distal Femoral Metaphysis

**Fig 5:**
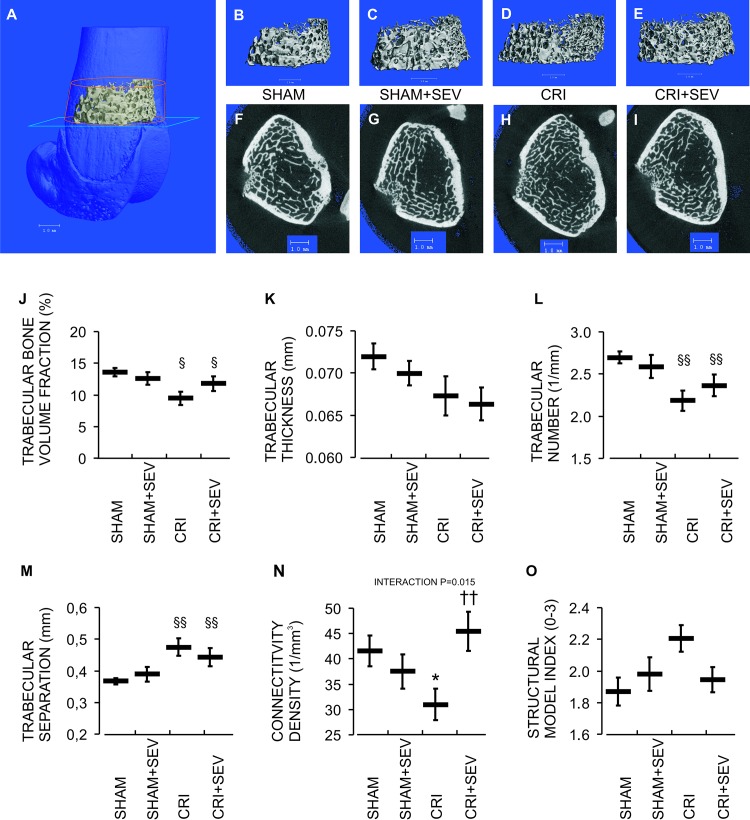
Trabecular bone at distal femoral metaphysis. (A) Scanned trabecular bone region inside the red cylinder; (B-E) trabecular bone structure and (F-I) cross-sectional image in each group, blue plane in image (A) depicts the location of images (F-I); (J) trabecular bone volume fraction; (K) trabecular thickness; (L) trabecular number; (M) trabecular separation, (N) connectivity density, (O) structural model index. Data denotations are mean (line) and SEM (whiskers); ^§^P<0.05, ^§§^P<0.01 CRI main effect; *P<0.05 vs. Sham; ^††^P<0.01 vs. CRI.

Fig 5A–5I visualize the microstructural changes (μCT) in the trabecular bone of the distal femoral metaphysis. Overall, the CRI-groups displayed a decrease in trabecular bone volume fraction (Fig 5J). More detailed evaluation showed a trend toward reduced trabecular thickness (*P* = 0.054, Fig 5K), and a decrease in trabecular number and increase in trabecular separation in CRI (Fig 5L–5M). Sevelamer-HCl treatment did not influence these variables, but prevented the CRI-induced decrease in trabecular connectivity density (Fig 5N). No significant differences were observed in the structural model index (Fig 5O).

## Discussion

We explored the effects of sevelamer-HCl treatment on femoral bone strength and structure in a model of advanced CRI. To mimic the clinical situation in renal disease, the first 14 weeks comprised a progression period of renal insufficiency and hyperphosphatemia, followed by a 9-week treatment period. The study protocol was successful, since characteristic plasma chemistry of advanced CRI was observed [23–25], and increased femoral bone fragility was detected. The detrimental changes in bone were effectively ameliorated by sevelamer-HCl, although the compound induced acidosis in both uremic and control rats.

Hyperphosphatemia is an essential factor for the development of CKD-MBD [1, 42], the treatment of which has been traditionally based on the use of oral calcium salts [43, 44]. However, phosphate binding with sevelamer has been found to provide significant clinical benefits when compared with calcium salts [8–10]. In haemodialysis patients, treatment with calcium carbonate, but not with sevelamer, was associated with decreased trabecular bone density in thoracic vertebrae [8]. In pediatric peritoneal dialysis patients, treatment with calcium carbonate or sevelamer resulted in equivalent control of phosphate, PTH and skeletal changes of secondary hyperparathyroidism. However, serum calcium levels and calcium x phosphate product increased with calcium carbonate, but not with sevelamer [9].

In rodent models of CRI, the harmful influence of hyperphosphatemia, and beneficial effect of sevelamer, on bone histology have been previously reported [12, 13, 45]. High phosphate intake reduced trabecular bone volume, irrespective of PTH levels, in the distal femur of 5/6 nephrectomized rats [45]. In adenine-induced CRI, sevelamer diet decreased osteoid volume, fibrosis volume, and cortical bone porosity in the femoral diaphysis [12]. In a murine model of metabolic syndrome with CKD and low bone turnover, sevelamer treatment showed several benefits: normalized serum phosphate and trabecular bone volume, increased osteoblast and osteoid surfaces, and increased bone formation rate [13].

Prompted by the above favorable influences of sevelamer on bone density and histology, and the fact that increased susceptibility to fractures is the most important clinical manifestation of metabolic bone disorders [1–3, 16, 19], we evaluated the functional integrity of bones using structural strength tests [17, 20]. This study focused on three regions of rat femur: 1) diaphysis at the midshaft, a tubular cortical bone structure, 2) neck, a tubular cortical structure with medullary trabeculae occupying approximately 7% of total bone volume [46], and 3) distal metaphysis which is mostly trabecular bone. In line with the above findings on bone histology, sevelamer ameliorated the CRI-induced loss of bone mineral, but also prevented the loss of structural strength of bones. In agreement with our previous studies [23, 24], the CRI-associated decrease in bone density was more prominent in the femoral neck (-11%) than midshaft (-3.4%), and the differences in bone density were inversely associated with changes in cross-sectional area in the femoral neck (sevelamer increased neck vBMD by 6% and reduced tCSA by -7% in rats with CRI) [23].

Given the well documented phosphate-binding effect of sevelamer-HCl [8, 9, 11–13, 27], our findings showing no differences in the *crude* plasma levels of phosphate and PTH between untreated and sevelamer-treated rats with CRI call for elaboration (Table 1). The absence of the phosphate-lowering effect can probably be attributed to the detrimental influence of sevelamer-HCl on the acid-base balance in rats with CRI. This influence was not due to selection bias, as the CRI groups were well matched: equal kidney tissue removal, and equal initial plasma creatinine, body weight, and urine output before the treatment. Sevelamer-HCl administration has been previously shown to increase dietary acid load and reduce serum bicarbonate levels [14, 15, 26]. Metabolic acidosis, in turn, enhances renal phosphate and calcium excretion probably due to net efflux of phosphate and calcium from bones [14, 47]. Accordingly, acidosis has a negative influence on physiology by predisposing to hyperphosphatemia, bone demineralization, and increased bone resorption [14, 15]. However, metabolic acidosis has also been reported to enhance the renal clearance of phosphate in both humans and 5/6 nephrectomized rats [47, 48]. Thus, acidosis *per se* may not alone explain the lack of the phosphate-lowering effect of sevelamer-HCl. The present results showed a numerically lower creatinine clearance without statistical significance (*P* = 0.059) in sevelamer-treated versus untreated rats with CRI. When the results were adjusted for the levels of plasma creatinine, the lowering effect of sevelamer-HCl on plasma phosphate and PTH in rats with CRI became apparent (Table 1). The observed strong correlation between plasma creatinine and phosphate concentrations suggests that the numerical differences in the levels of renal function, albeit statistically insignificant, may explain the lack of the reduction of plasma crude phosphate concentrations in the sevelamer-HCl treated rats. At the time when the study was conducted only sevelamer-HCl could be provided by the manufacturer for the present experimental study.

We observed reduced plasma level of 1,25(OH)_2_D_3_ after the sevelamer-HCl diet (P<0.001), an effect that is not explained by differences in renal function, or plasma concentrations of phosphate, PTH, and FGF-23 [27]. It is important to notice that 1,25(OH)_2_D_3_ was reduced even in the sham-operated control rats. The plasma 25OH-D_3_ concentrations were also reduced in the Sham+Sev (-20%) and CRI+Sev (-33%) groups when compared with respective controls. One possibility for these changes is reduced chow intake in the sevelamer-HCl groups, but unchanged weight gain does not support this notion. Sevelamer also interferes with the absorption of fat-soluble vitamins in the gut [49, 50], and this mechanism may partially explain the decreased plasma calcidiol in the Sham+Sev and CRI+Sev rats. However, it seems unlikely that moderate reductions in plasma 25OH-D_3_ could explain the far greater reductions in 1,25(OH)_2_D_3_ following sevelamer-HCl therapy (Sham+Sev -64%, CRI+Sev -52%). Since acidosis is known to inhibit 1,25(OH)_2_D_3_ synthesis in the rat [51], reduced pH remains the most likely explanation for the reduced plasma 1,25(OH)_2_D_3_ concentrations following sevelamer-HCl treatment.

Although treatment with sevelamer-HCl has been reported to reduce plasma FGF-23 concentrations in the adenine-induced model of severe CRI [27], sevelamer did not reduce plasma FGF-23 in the present study. Previously, metabolic acidosis has been reported to directly increase FGF-23 production in mouse bone [52]. Therefore, acidosis provides a potential explanation for the lack of changes in plasma FGF-23 concentrations in the sevelamer-treated rats. High FGF-23 concentration may also have a potential role in the observed beneficial biomechanical changes of bone in the sevelamer-treated rats, since FGF-23 has been reported to influence bone mineralization independently of systemic phosphate homeostasis [53]. In dialyzed pediatric patients, high levels of FGF-23 were associated with improved indices of skeletal mineralization [54]. In adult hemodialysis patients, FGF-23 was an independent predictor of bone mineralization, so that mineralization lag time was abnormally long in patients with moderately elevated FGF-23 concentration (<2,000 pg/mL), but was normal in patients with high levels of FGF-23 [55]. Although we cannot rule out the possibility that the beneficial effects of sevelamer-HCl on bone were partially mediated via high levels of FGF-23, the finding that plasma FGF-23 concentrations did not differ between the CRI versus CRI+Sev groups argues against this view.

Like in the clinical setting, mortality in advanced experimental CRI is high [24, 25]. Mortality rates exceeding 80% have been reported in 5/6 nephrectomized rats that were followed for 6 months [22]. In order to avoid the complication that most severely affected rats would have been lost from the analysis, a protocol with predefined endpoints for premature termination was applied. This protocol was effective, as the renal insufficiency was clearly more advanced in the prematurely sampled rats than in those rats that went through the whole treatment period (S1 Table). We want to stress that the average length of the treatment period (50 days) was equal in the untreated and sevelamer-treated rats with CRI. The present experiments did not include analyses of bone histology, because dynamic histomorphometry would not have been possible in all animals, since the prematurely sampled rats would have been lost from bone labeling. In addition, dynamic histomorphometry would rather have reflected the 5 days preceding the final sampling, i.e. a period when the indices of renal function were numerically lower in the sevelamer-treated than untreated rats with CRI. In contrast, the preserved bone strength can be argued to more adequately reflect the influence of the whole 9-week treatment period on bone.

In summary, the phosphate binder sevelamer-HCl effectively prevented the experimental CRI-induced changes in femoral bone mineral density and breaking load, in spite of advanced renal insufficiency and acidosis. In healthy control rats, sevelamer administration was without any major effects on bone, although plasma 25OH-D_3_ and 1,25-(OH)2D3 concentrations were clearly reduced. These experimental results suggest that sevelamer treatment can efficiently preserve the mechanical competence of bone in CRI. Importantly, the present beneficial effects on bones were not explained by influences of sevelamer-HCl on renal function.

## Supporting Information

S1 File Original data. (PDF)

S2 File Original data. (PDF)

S1 Table Analyses from prematurely sampled rats versus those gone through the whole treatment period. (DOC)
