# Supplementary material for: Phosphate Binding with Sevelamer Preserves Mechanical Competence of Bone Despite Acidosis in Advanced Experimental Renal Insufficiency
Source: PLoS One. 2016 Sep 22;11(9):e0163022. doi: 10.1371/journal.pone.0163022 (PMC5033583; doi:10.1371/journal.pone.0163022)
Supplement: S1 File — (PDF) [file pone.0163022.s001.pdf]

Gr Code	RatNo	Th length	KidRem_g/kg	Wt	Blood pH	Hb	P-Urea mM	P-Ca mM	P-Krea uM	P-Pi mM	P-D25 nM	P-D1,25 pM	P-FGF-23	P-PTH pg/ml
NX	1	54	6,15	483	7,24	156	13,96	2,30	90,70	1,90	51,50	47,52	2316,00	1340,57
NX	2	54	5,76	408	7,11	128	39,50	2,14	158,80	4,24	42,94	27,42	18530,00	3678,62
NX	4	34	6,91	636	7,14		19,93	2,33	82,80	1,92	35,15	48,83	1496,00	489,16
NX	5	54	6,90	480	7,33	157	11,24	2,28	90,70	1,68	33,47	100,47	1856,00	493,34
NX	8	38	6,16	388	6,96		100,73	1,73	563,80	9,82	62,40	11,60	14070,00	2268,86
NX	10	51	6,69	366										
NX	12	48	5,65	350										
NX	13	59	6,34	499	7,40	168	14,52	2,27	100,70	1,74	41,94	117,65	1750,00	622,57
NX	14	59	6,57	514	7,46	164	13,66	2,44	76,20	1,72	35,15	82,13	2781,00	426,61
NX	15	22	6,50	288	6,85		49,60	2,30	163,80	5,62	33,27	17,67	32860,00	2522,22
NX	16	59	5,81	503	7,54	174	9,18	2,34	64,20	1,33	63,07	204,66	793,30	164,69
NX	19	60	6,47	487	7,31	151	13,34	2,53	77,80	1,92	25,93	100,45	3523,00	863,96
NX	20	60	6,93	507	7,39	148	10,61	2,42	62,70	1,47	36,04	178,57	1173,00	33,79
Se-NX	2	54	6,52	500	7,29	166	13,67	2,36	87,80	1,14	28,12	125,89	549,80	4,60
Se-NX	3	26	6,68	318	6,88		115,86	2,21	547,30	9,66	22,51	7,88	89780,00	2219,05
Se-NX	4	51	5,97	497										
Se-NX	5	53	6,62	336	7,21		52,33	2,57	178,10	2,10	21,83	8,27	5796,00	229,92
Se-NX	6	54	5,99	492	7,07	173	7,52	2,43	66,30	1,48	24,01	148,41	768,30	23,56
Se-NX	7	54	6,31	465	6,97	151	26,36	2,50	156,60	2,04	29,89	34,74	910,70	231,27
Se-NX	8	47	5,91											
Se-NX	9	57	5,82	443	7,03	117	31,54	2,45	166,70	3,07	20,59	4,39	6640,00	698,41
Se-NX	10	58	6,97	463	7,27	144	23,29	2,38	145,90	1,88	28,64	79,76	770,30	372,71
Se-NX	11	57	6,90	402	7,06	110	65,88	2,40	407,50	2,89	23,13	6,92	4494,00	351,21
Se-NX	12	58	5,57	497	7,28	146	15,54	2,56	103,60	1,72	29,92	17,92	1009,00	74,06
Se-NX	13	51	6,09	343			112,26	2,54	902,60	11,67	48,48	5,51	145200,00	3266,38
Se-NX	14	38	6,62	494	7,15		39,33	2,28	143,00	2,71	30,85	9,37	3744,00	576,34
Sham	1	60		537			6,50	2,32	50,50	1,68	46,78	434,82	748,20	131,06
Sham	2	62		450	7,49	183	6,94	2,29	57,70	1,00	55,16	455,27	795,70	80,68
Sham	3	62		461		176	6,40	2,30	72,00	1,45	46,08	407,52	870,70	177,48
Sham	4	62		507	7,56	183	6,68	2,46	47,70	1,43	77,93	443,08	772,40	35,75
Sham	5	62		556	7,48	178	7,48	2,32	48,40	1,26	51,02	351,73	784,70	17,37
Sham	6	63		418	7,38	168	6,77	2,41	48,40	1,05	62,87	448,62	714,20	30,50
Sham	7	63		477	7,47	161	5,26	2,45	36,20	1,12	59,18	469,52	607,10	2,61
Sham	8	63		520	7,26	180	5,58	2,27		1,06	60,49	385,65	732,30	28,47
Sham	9	63		437	7,18	181	6,95	2,31	40,50	1,29	67,83	498,78	683,40	26,21
Sham	14	66		477	7,23	175	9,24	2,29	48,40	1,18	44,57	324,67	751,30	16,22
Se-Sh	1	62		503	7,46	189	7,51	2,29	44,80	1,22	41,51	126,69	675,60	25,84
Se-Sh	5	63		475	7,07	179	6,23	2,24	47,70	1,43	30,97	119,80	668,70	204,19
Se-Sh	9	63		464										
Se-Sh	10	63		491	7,27	185	5,98	2,27	58,40	1,38	64,79	168,16	692,00	219,85
Se-Sh	11	63		491	6,92	175	7,42	2,31	64,90	1,66	43,36	161,56	788,00	168,15
Se-Sh	12	63		499	7,16	185	7,19	2,34	47,00	1,55	56,23	186,24	753,10	108,10
Se-Sh	14	66		495	7,42	183	7,21	2,50	60,60	1,43	35,66	146,85	771,20	8,34
Se-Sh	15	66		650	7,31	177	9,10	2,40	36,90	1,57	45,40	146,47	523,60	7,39
