# Supplementary material for: Phosphate Binding with Sevelamer Preserves Mechanical Competence of Bone Despite Acidosis in Advanced Experimental Renal Insufficiency
Source: PLoS One. 2016 Sep 22;11(9):e0163022. doi: 10.1371/journal.pone.0163022 (PMC5033583; doi:10.1371/journal.pone.0163022)
Supplement: S2 File — (PDF) [file pone.0163022.s002.pdf]

Femur Midshaft

Group	Gr Code	Femur	BW	Length	cBMD	tCSA	cCSA	Cortex-AP	Cortex-ML	Fmax-AP	Fmax-ML
CRI	NX	R	483	40,70	1428,4	14,12	7,16	0,808	0,926	153,4	
CRI	NX	R	408	40,80	1349,8	15,63	9,23	0,950	1,021	174,6	
CRI	NX	R	636	40,41	1440,4	15,25	8,31	0,950	1,045	191,4	
CRI	NX	R	480	40,30	1456,0	13,50	7,70	0,974	0,926	175,1	
CRI	NX	R	388	40,60	1372,5	15,61	8,23	0,950	0,950	174,4	
CRI	NX	R	366	40,51	1430,0	14,19	7,80	0,879	1,021	82,4	
CRI	NX	R	350	40,04	1339,2	14,58	8,16	0,998	0,950	119,3	
CRI	NX	R	499	39,66	1432,4	13,97	7,67	0,950	0,926	154,5	
CRI	NX	R	514	42,24	1405,3	15,14	8,40	1,021	0,879	191,8	
CRI	NX	R	288	38,80	1391,6	12,41	6,12	0,760	0,903	72,8	
CRI	NX	R	503	41,95	1449,1	14,47	7,85	0,879	0,950	190,6	
CRI	NX	R	487	40,51	1427,0	15,17	7,65	0,831	0,902	166,8	
CRI	NX	R	507	42,31	1441,3	13,99	8,08	0,855	0,997	181,6	
CRI	NX	L	483	41,26	1426,9	14,28	7,37	0,808	0,974		159,8
CRI	NX	L	408	41,12	1348,2	14,93	8,96	0,993	1,021		148,6
CRI	NX	L	636	40,88	1432,3	14,81	7,99	0,879	1,021		183,9
CRI	NX	L	480	40,34	1440,3	13,47	7,78	0,903	0,974		168,9
CRI	NX	L	388	40,58	1381,4	15,00	8,21	0,926	0,997		167,9
CRI	NX	L	350	40,12	1354,3	14,95	7,91	0,926	0,902		154,8
CRI	NX	L	499	39,79	1439,9	13,45	7,43	0,879	0,902		160,1
CRI	NX	L	514	42,12	1422,3	15,34	8,73	1,045	0,950		182,8
CRI	NX	L	288	38,99	1378,8	12,25	6,49	0,879	0,950		140,1
CRI	NX	L	503	42,10	1457,8	14,52	8,04	0,903	0,926		176,4
CRI	NX	L	487	40,74	1435,4	15,04	7,37	0,760	0,712		155,4
CRI	NX	L	507	41,85	1438,8	14,51	8,37	0,974	0,974		183,5
CRI+Sev	Se-NX	R	500	41,67	1460,0	14,51	9,02	1,045	1,045	228,0	
CRI+Sev	Se-NX	R	318	40,15	1410,9	14,77	7,68	0,902	0,926	183,1	
CRI+Sev	Se-NX	R	497	40,78	1443,6	13,87	8,01	0,479	0,926	199,2	
CRI+Sev	Se-NX	R	336	38,07	1402,1	12,54	6,14	0,855	0,831	133,9	
CRI+Sev	Se-NX	R	492	40,25	1454,8	13,49	8,10	0,974	1,021	172,5	
CRI+Sev	Se-NX	R	465	39,72	1435,1	12,38	7,17	0,950	0,903	168,1	
CRI+Sev	Se-NX	R	501	40,74	1439,3	13,99	7,91	0,926	1,021	191,9	
CRI+Sev	Se-NX	R	443	40,57	1440,9	13,98	8,21	0,950	1,069	169,5	
CRI+Sev	Se-NX	R	463	40,57	1435,8	13,94	7,88	0,950	0,974	180,6	
CRI+Sev	Se-NX	R	402	39,62	1435,8	14,07	7,60	0,903	0,926	185,7	
CRI+Sev	Se-NX	R	497	40,85	1436,5	15,07	9,06	1,021	1,069	206,6	
CRI+Sev	Se-NX	R	343	39,27	1422,6	14,35	6,53	0,760	0,808	147,9	
CRI+Sev	Se-NX	R	494	42,87	1432,3	17,80	10,89	1,259	1,092	274,8	
CRI+Sev	Se-NX	L	500	41,49	1473,0	14,35	9,02	1,140	1,069		202,8
CRI+Sev	Se-NX	L	318	40,03	1412,2	14,16	7,24	0,855	0,926		170,3
CRI+Sev	Se-NX	L	497	40,75	1452,5	14,03	8,04	0,998	0,855		178,7
CRI+Sev	Se-NX	L	336	38,12	1423,7	12,15	6,12	0,831	0,855		115,1
CRI+Sev	Se-NX	L	492	40,79	1455,7	13,51	7,98	0,903	1,021		186,9
CRI+Sev	Se-NX	L	465	39,39	1451,7	12,53	7,19	0,855	1,021		135,3
CRI+Sev	Se-NX	L	501	40,50	1462,3	13,99	8,15	0,926	0,974		196,8
CRI+Sev	Se-NX	L	443	40,44	1454,4	13,74	8,14	0,974	1,188		188,2
CRI+Sev	Se-NX	L	463	40,20	1440,9	13,91	7,81	0,879	1,021		158,6
CRI+Sev	Se-NX	L	402	40,01	1407,5	14,32	7,72	0,998	0,879		147,7
CRI+Sev	Se-NX	L	497	41,34	1439,3	14,59	8,88	0,950	1,069		199,2
CRI+Sev	Se-NX	L	343	39,46	1439,9	13,73	6,77	0,879	0,879		146,8
CRI+Sev	Se-NX	L	494	42,85	1440,5	16,94	10,47	1,164	1,164		249,7

Femur Midshaft

Group	Gr Code	Femur	BW	Length	cBMD	tCSA	cCSA	Cortex-AP	Cortex-ML	Fmax-AP	Fmax-ML
Sham	Sham	R	537	41,2	1454,7	14,82	8,43	0,903	1,021	211,7	
Sham	Sham	R	450	38,37	1464,2	13,23	7,84	0,903	1,060	178,8	
Sham	Sham	R	461	39,91	1471,0	14,23	7,91	0,950	1,021	199,6	
Sham	Sham	R	507	41,63	1463,0	15,05	8,89	1,045	1,069	213,9	
Sham	Sham	R	556	41,71	1453,5	15,52	9,15	1,021	1,045	225,7	
Sham	Sham	R	418	39,9	1463,4	12,82	7,21	0,879	0,974	161,8	
Sham	Sham	R	477	41,36	1462,9	14,21	8,07	0,950	1,021	180,5	
Sham	Sham	R	520	41,48	1447,2	15,20	8,37	0,950	0,902	214,0	
Sham	Sham	R	437	39,94	1462,3	12,90	7,60	0,974	1,021	173,2	
Sham	Sham	R	477	41,3	1450,8	15,29	8,32	0,903	0,974	154,4	
Sham	Sham	L	537	41,41	1462,9	14,54	8,37	0,950	1,021		205,0
Sham	Sham	L	450	38,51	1484,8	13,33	7,60	0,927	1,092		176,9
Sham	Sham	L	461	40,24	1463,8	14,31	7,93	0,879	1,045		194,0
Sham	Sham	L	507	40,99	1457,6	14,79	8,58	0,997	0,974		210,6
Sham	Sham	L	556	41,88	1455,7	15,68	8,78	0,902	1,093		249,3
Sham	Sham	L	418	40,1	1478,3	12,75	7,15	0,808	1,045		162,2
Sham	Sham	L	477	41,19	1487,1	13,62	7,62	0,922	0,998		163,9
Sham	Sham	L	520	41,6	1458,1	14,94	8,19	0,926	1,021		197,7
Sham	Sham	L	437	40,25	1459,2	12,63	7,46	0,950	0,997		178,7
Sham	Sham	L	477	41,05	1456,6	15,43	8,20	0,855	1,045		199,2
Sham+Sev	Se-Sh	R	503	40,61	1476,0	14,66	8,24	0,950	1,069	209,5	
Sham+Sev	Se-Sh	R	475	40,09	1462,8	13,55	7,97	0,974	1,045	201,3	
Sham+Sev	Se-Sh	R	464	41,14	1444,4	14,17	7,76	0,974	0,998	175,5	
Sham+Sev	Se-Sh	R	491	41,7	1463,0	14,95	9,06	1,069	1,045	227,8	
Sham+Sev	Se-Sh	R	491	40,47	1465,9	14,60	8,33	0,950	1,045	202,8	
Sham+Sev	Se-Sh	R	499	40,59	1459,6	13,72	8,24	1,045	1,045	176,0	
Sham+Sev	Se-Sh	R	495	41,37	1464,6	14,92	8,76	1,045	1,093	184,6	
Sham+Sev	Se-Sh	R	650	39,9	1461,0	14,07	8,16	0,998	0,997	197,8	
Sham+Sev	Se-Sh	L	503	40,64	1477,7	14,62	8,28	0,927	1,093		210,3
Sham+Sev	Se-Sh	L	464	41,15	1461,7	13,58	7,36	0,855	1,021		164,2
Sham+Sev	Se-Sh	L	491	41,56	1471,3	14,83	8,94	0,926	1,092		205,4
Sham+Sev	Se-Sh	L	491	40,43	1472,8	14,29	8,19	0,927	1,069		189,8
Sham+Sev	Se-Sh	L	499	40,14	1472,0	13,46	7,71	0,950	1,069		169,3
Sham+Sev	Se-Sh	L	495	41,49	1471,2	14,52	8,77	1,045	1,069		205,4
Sham+Sev	Se-Sh	L	650	40,18	1466,8	14,05	8,00	0,903	0,998		179,4

Femur neck

Group	Gr Code	Femur	vBMD	tCSA	tBMC	Fmax-FN	Dia_ML	Dia_CC
CRI	NX	R	1023,5	4,88	4,99	134,9	2,33	3,34
CRI	NX	R	827,8	6,57	5,44	138,3	2,13	2,77
CRI	NX	R	864,9	6,63	5,73	142,1	2,36	3,55
CRI	NX	R	1085,0	4,98	5,41	132,2	2,23	2,86
CRI	NX	R	1024,3	5,02	5,14	127,8	2,20	2,68
CRI	NX	R	885,8	5,77	5,11	116,9	2,30	3,40
CRI	NX	R	899,1	5,98	5,37	107,1	2,09	3,42
CRI	NX	R	938,5	6,30	5,91	134,4		
CRI	NX	R	1113,0	4,88	5,43	143,5	2,37	3,37
CRI	NX	R	888,2	5,30	4,70	121,8	2,10	2,65
CRI	NX	R	1105,2	4,83	5,33	122,7	2,06	2,40
CRI	NX	R	1003,9	5,14	5,17	128,9	2,38	2,72
CRI	NX	R	1103,1	4,90	5,40	136,9	2,20	3,15
CRI	NX	L	1003,7	5,48	5,50	125,8		
CRI	NX	L	954,5	5,40	5,16	152,7		
CRI	NX	L	926,0	5,71	5,29	161,3		
CRI	NX	L	1089,3	4,98	5,43	145,2		
CRI	NX	L	1028,7	5,33	5,48	170,0		
CRI	NX	L	803,2	7,83	6,29	109,2		
CRI	NX	L	907,5	6,10	5,54	150,9		
CRI	NX	L	1058,8	5,33	5,64	158,8		
CRI	NX	L	880,1	4,87	4,29	97,8		
CRI	NX	L	1092,0	5,27	5,76	206,6		
CRI	NX	L	1005,0	5,12	5,14	163,7		
CRI	NX	L	1074,6	4,97	5,34	110,6		
CRI+Sev	Se-NX	R	1083,0	4,87	5,27	130,2	2,19	2,89
CRI+Sev	Se-NX	R	970,2	5,22	5,07	137,9	2,27	3,07
CRI+Sev	Se-NX	R	1072,7	4,90	5,25	125,2	2,29	2,89
CRI+Sev	Se-NX	R	941,6	5,83	5,49	*90,15	2,20	3,23
CRI+Sev	Se-NX	R	1127,6	4,65	5,25	143,6	2,18	2,59
CRI+Sev	Se-NX	R	1000,3	5,46	5,46	159,2	2,07	2,90
CRI+Sev	Se-NX	R	1058,6	5,14	5,44	116,9	2,10	2,77
CRI+Sev	Se-NX	R	1044,8	5,01	5,24	112,2	2,07	2,75
CRI+Sev	Se-NX	R	1036,1	5,57	5,77	164,4	2,26	2,86
CRI+Sev	Se-NX	R	1071,0	4,52	4,84	129,6	2,09	3,21
CRI+Sev	Se-NX	R	1075,9	5,26	5,66	170,5	2,23	2,84
CRI+Sev	Se-NX	R	946,8	4,78	4,52	108,1	2,07	2,77
CRI+Sev	Se-NX	R	1156,6	4,82	5,57	145,1	2,25	2,83
CRI+Sev	Se-NX	L	1070,9	4,77	5,11	124,2		
CRI+Sev	Se-NX	L	996,6	5,32	5,30	182,5		
CRI+Sev	Se-NX	L	1051,9	5,61	5,90	190,2		
CRI+Sev	Se-NX	L	903,1	5,70	5,15	105,6		
CRI+Sev	Se-NX	L	1094,0	5,05	5,52	139,5		
CRI+Sev	Se-NX	L	979,0	5,26	5,15	*88,61		
CRI+Sev	Se-NX	L	1055,8	5,07	5,35	160,3		
CRI+Sev	Se-NX	L	1053,4	5,00	5,26	138,3		
CRI+Sev	Se-NX	L	1031,6	5,22	5,38	161,5		
CRI+Sev	Se-NX	L	1106,6	4,45	4,92	119,3		
CRI+Sev	Se-NX	L	1057,2	5,33	5,64	176,5		
CRI+Sev	Se-NX	L	958,7	5,18	4,97	139,6		
CRI+Sev	Se-NX	L	1135,6	4,86	5,51	152,3		

Femur neck

Group	Gr Code	Femur	vBMD	tCSA	tBMC	Fmax-FN	Dia_ML	Dia_CC
Sham	Sham	R	1180,0	4,68	5,52	127,7	2,17	2,69
Sham	Sham	R	1105,2	4,65	5,13	133,1	2,06	2,83
Sham	Sham	R	1120,6	4,98	5,58	148,9	2,15	2,81
Sham	Sham	R	1078,7	5,77	6,23	141,6	2,28	2,93
Sham	Sham	R	1090,3	5,77	6,29	165,4	2,30	3,13
Sham	Sham	R	1073,1	4,86	5,21	121,0	2,20	2,69
Sham	Sham	R	1094,2	5,27	5,76	164,2	2,20	2,88
Sham	Sham	R	1040,2	6,19	6,44	167,5	2,18	3,22
Sham	Sham	R	1149,2	4,54	5,21	103,8	2,10	2,60
Sham	Sham	R	1117,6	5,34	5,97	144,2	2,22	2,86
Sham	Sham	L	1148,0	4,96	5,70	160,9		
Sham	Sham	L	1160,9	4,57	5,30	144,2		
Sham	Sham	L	1150,0	4,97	5,72	175,8		
Sham	Sham	L	1041,6	5,99	6,24	213,7		
Sham	Sham	L	1085,0	6,35	6,89	214,5		
Sham	Sham	L	1090,1	4,65	5,07	147,3		
Sham	Sham	L	1111,2	4,84	5,37	173,9		
Sham	Sham	L	1065,0	5,55	5,91	148,2		
Sham	Sham	L	1137,3	4,41	5,02	123,7		
Sham	Sham	L	1054,8	5,50	5,80	165,0		
Sham+Sev	Se-Sh	R	1064,6	5,57	5,93	152,2	2,05	2,71
Sham+Sev	Se-Sh	R	1120,2	4,94	5,54	146,7	2,18	2,70
Sham+Sev	Se-Sh	R	1063,1	5,16	5,49	132,0	2,24	3,11
Sham+Sev	Se-Sh	R	1081,2	5,19	5,62	142,1	2,08	3,01
Sham+Sev	Se-Sh	R	1084,5	4,82	5,22	127,7	2,04	2,56
Sham+Sev	Se-Sh	R	1178,1	4,55	5,36	112,4	2,10	2,64
Sham+Sev	Se-Sh	R	1131,9	4,68	5,30	143,5	2,18	2,76
Sham+Sev	Se-Sh	R	1040,5	5,92	6,16	142,4	2,31	3,04
Sham+Sev	Se-Sh	L	1002,4	6,17	6,19	126,5		
Sham+Sev	Se-Sh	L	1021,6	5,36	5,47	121,9		
Sham+Sev	Se-Sh	L	1110,4	5,12	5,69	161,1		
Sham+Sev	Se-Sh	L	1049,4	5,39	5,66	192,3		
Sham+Sev	Se-Sh	L	1048,0	4,74	4,97	113,8		
Sham+Sev	Se-Sh	L	1064,2	5,49	5,85	149,2		
Sham+Sev	Se-Sh	L	1022,0	6,79	6,94	196,1		
