# Supplementary material for: Phosphate Binding with Sevelamer Preserves Mechanical Competence of Bone Despite Acidosis in Advanced Experimental Renal Insufficiency
Source: PLoS One. 2016 Sep 22;11(9):e0163022. doi: 10.1371/journal.pone.0163022 (PMC5033583; doi:10.1371/journal.pone.0163022)
Supplement: S1 Table — (DOC) [file pone.0163022.s003.doc]

**S1 Supplementary Table 1. Analyses from rats with chronic renal insufficiency: prematurely sampled rats versus those gone through the whole treatment period.**

|  | Whole treatment period  n=15 | Premature sampling  n=11 |
| --- | --- | --- |
| Animal weight (g) |  |  |
| Before treatment | 440±6 | 452±17 |
| Final | 480±9 | 402±34^*^ |
| Creatinine (µmol/l) | 119±21 | 369±116^**^ |
| Urea (mmol/l) | 19.8±3.8 | 70.0±14.6^***^ |
| Phosphate (mmol/l) | 2.0±0.2 | 6.2±1.6^**^ |
| Calcium (mmol/l) | 2.38±0.03 | 2.17±0.11^*^ |
| PTH (pg/ml)^1^ | 594±224 | 1653±452^*^ |
| 1,25(OH)_2_D_3_ (pmol/l) | 107±26 | 16±6^**^ |
| FGF-23 (pg/ml)^1^ | 3038±1114 | 24624±13844^**^ |
| Blood pH | 7.25±0.04 | 7.03±0.06^*^ |
|  |  |  |

Results are mean±SE; ^1^statistics from log-transformed values;
^*^P<0.05, ^*^P<0.01, ^*^P<0.001 prematurely sampled rats vs. rats gone through the whole treatment period before sampling.
